# Reduced heparan sulfate levels in cerebrospinal fluid reflect brain neuron correction in Sanfilippo B mice

**DOI:** 10.1172/JCI195268

**Published:** 2025-08-28

**Authors:** Steven Q. Le, Alexander Sorensen, Soila Sukupolvi, Gianna Jewhurst, Grant Austin, Balraj Doray, Jonathan D. Cooper, Patricia I. Dickson

**Affiliations:** Department of Pediatrics, Washington University School of Medicine, St. Louis, Missouri, USA.

**Keywords:** Genetics, Neuroscience, Biomarkers, Lysosomes, Neurological disorders

## Abstract

A biomarker in spinal fluid reflects brain disease in a rare genetic disorder, with implications for how we diagnose and monitor the disease.

**To the Editor:** Mucopolysaccharidoses (MPS) are a group of inherited, lysosomal diseases caused by deficiency of enzymes critical for the catabolism of glycosaminoglycans. Heparan sulfate (HS) glycosaminoglycans accumulate in several types of MPS, including Sanfilippo syndrome (MPS III), Hunter syndrome (MPS II), Hurler syndrome (MPS I), and Sly syndrome (MPS VII). Cerebrospinal fluid HS (CSF HS) has been considered as a feasible biomarker for ascertaining brain HS levels in MPS disorders (1). Ideally, its measurement could be used to determine the response to brain-directed therapy in clinical trials for patients with MPS. Preclinical and clinical studies demonstrate a correlation of brain HS and CSF HS, suggesting a relationship (2). However, because HS is ubiquitous, it has been difficult to determine whether CSF HS truly reflects brain HS levels. The observation that CSF HS is lowered following intravenous enzyme replacement therapy (which largely does not cross the blood-brain barrier [BBB]) suggests the possibility that HS may cross from blood into CSF (2).

Experiments designed to test this hypothesis must prevent the cross-correction that occurs with secreted, soluble lysosomal enzymes. To this end, using published methods (3), we generated an expression construct with the *NAGLU* gene encoding α-N-acetylglucosaminidase (the enzyme deficient in MPS IIIB), a 6-glycine linker, and a 114-bp nucleotide segment corresponding to the transmembrane region and cytosolic tail of *LAMP1,* which encodes the lysosomal-associated membrane protein-1, followed by a c-Myc epitope tag after the cytosolic tail. To test expression in vitro, human MPS IIIB fibroblasts were transduced with lentiviral NAGLU-LAMP1 (Supplemental Methods; supplemental material available online with this article; https://doi.org/10.1172/JCI195268DS1). Cells and media were harvested and assayed for NAGLU activity as described previously (4). We confirmed intracellular NAGLU activity but found no NAGLU activity in the secreted media, consistent with successful membrane tethering (Supplemental Figure 1). We then performed in vivo testing in *Naglu^–/–^* mice. To deliver NAGLU-LAMP1 systemically but avoid treating brain disease, we used adeno-associated viral vector-7 (AAV7), which does not cross the BBB when administered intravenously. We delivered 1.5 × 10^11^ vector genomes per mouse of AAV7-NAGLU-LAMP1 by tail vein at 4 weeks of age, avoiding the neonatal period during which the BBB is not completely intact. Four weeks after dose administration, NAGLU activity was detected in systemic bodily organs but not in the brain (Figure 1A)**,** with a concomitant decrease in β-hexosaminidase (β-Hex) activity in the organs showing good NAGLU activity (Supplemental Figure 2A). Consistent with lack of secretion of the membrane-tethered enzyme, NAGLU activity was not detected in serum (Figure 1A). Brain homogenates, serum, and CSF were assayed for HS by glycan reductive isotope labeling liquid chromatography/mass spectrometry (GRIL-LC/MS) (5, 6). Serum HS, but not brain or CSF HS, was reduced in treated *Naglu^–/–^* mice compared with untreated controls (Figure 1, B–D).

Next, we designed a separate experiment to study the effect on CSF HS when NAGLU restoration is largely confined to brain neurons. For this, we used an AAV9 vector with NAGLU-LAMP1 expression under the control of a synapsin-1 promoter. We administered 6.5 × 10^10^ vector genomes (high dose) or 6.5 × 10^9^ vector genomes (low dose) per mouse of AAV9-Syn1-NAGLU-LAMP1 into one lateral cerebral ventricle of *Naglu*^–/–^ mice at P1 or P2. Four weeks after dose administration, NAGLU activity was readily detected in brains of mice treated with both vector doses, while NAGLU activity in the periphery, including heart, kidney, liver, and serum, was undetectable (low dose) or extremely low (high dose) (Figure 1E). As before, β-Hex activity was only significantly decreased in the organs expressing NAGLU (Supplemental Figure 2B). Confocal microscopy confirmed neuronal expression and distribution of NAGLU-LAMP1 (Supplemental Figure 3). We also observed a decreased neuroimmune response, as indicated by significantly reduced staining for markers of microglial activation and astrocytosis in the brains of treated *Naglu*^–/–^ mice, supporting a therapeutic effect (Supplemental Figure 4). GRIL-LC/MS showed that brain HS was normalized and CSF HS was reduced in the mice that were treated intracerebroventricularly with AAV9-Syn1-NAGLU-LAMP1, whereas there was no difference in serum HS between treated and untreated *Naglu^–/–^* mice (Figure 1, F–H).

These results, summarized in Figure 1I, suggest that CSF HS reflects HS in the central nervous system. Moreover, as CSF HS was normalized with restoration of NAGLU activity in brain neurons, the results support the utility of CSF HS as a therapeutic biomarker for gauging improvement in brain disease due to MPS.

For detailed methods, information regarding sex as a biological variable, statistics, study approval, author contributions, and acknowledgments, see the supplemental materials.

## Funding support

This work is the result of NIH funding, in whole or in part, and is subject to the NIH Public Access Policy. Through acceptance of this federal funding, the NIH has been given a right to make the work publicly available in PubMed Central.

- NIH 1RM1 NS132962 (to PID and JDC).
- NIH 1R38 AI174266 (to GA).
- Hope Center Viral Vectors Core at Washington University School of Medicine.

## Supplementary Material

Supplemental data

Supporting data values

## Figures and Tables

**Figure 1:**
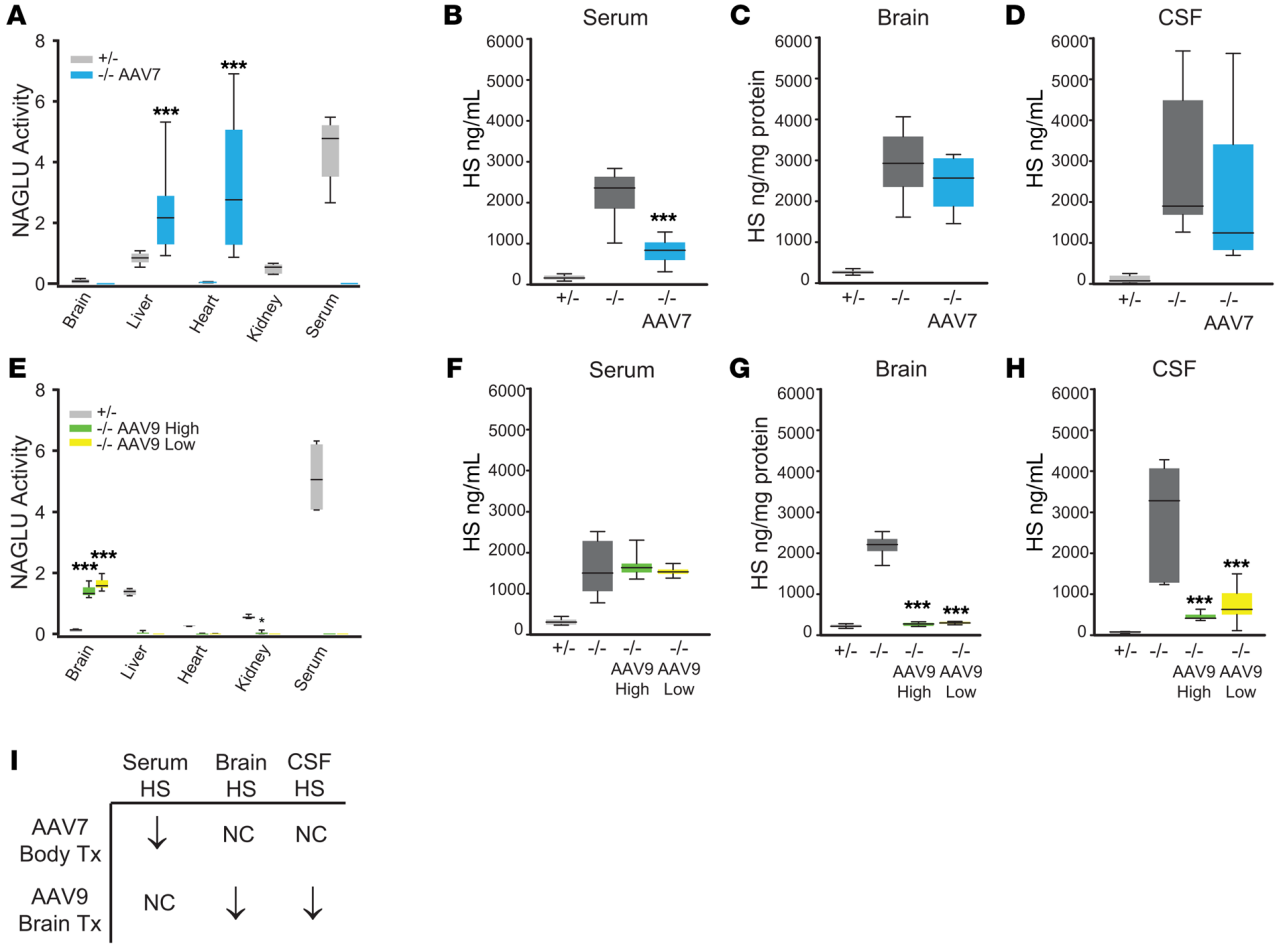
NAGLU activity and HS. (**A**) NAGLU enzymatic activity in 8-week-old *Naglu*^–/–^ mice (*n* = 12; 8 females, 4 males) treated at 4 weeks with AAV7-NAGLU-LAMP1 (AAV7) intravenously, compared with untreated *Naglu^–/–^* mice (*n* = 10: 5 females, 5 males) and *Naglu^+/–^* mice (*n* = 10; 5 females, 5 males). Horizontal lines represent means. *Naglu*^–/–^ mice have undetectable NAGLU activity (data not shown). (**B**–**D**) Total HS in serum, brains, and CSF of mice treated with AAV7-NAGLU-LAMP1 and controls. Box plots depict median (line), upper and lower quartiles (box bounds), and 10th and 90th centiles (whiskers). (**E**) NAGLU enzymatic activity in 4-week-old *Naglu*^–/–^ mice treated intracerebroventricularly at P1 or P2 with 6.5 × 10^10^ vector genomes (AAV9-High; *n* = 13; 3 females, 10 males) or 6.5 × 10^9^ vector genomes (AAV9-Low; *n* = 8: 4 females, 4 males) per mouse of AAV9-Syn1-NAGLU-LAMP1, compared with untreated *Naglu^–/–^* mice (*n* = 8; 2 females, 6 males) and *Naglu^+/–^* controls (*n* = 7; 3 females, 4 males). Horizontal lines represent means. *Naglu*^–/–^ mice have undetectable NAGLU activity (data not shown). (**F**–**H**) Total HS in serum, brains, and CSF of the mice treated with AAV9-Syn1-NAGLU-LAMP1 and controls. Box plots depict median (line), upper and lower quartiles (box bounds), and 10th and 90th centiles (whiskers). *P < 0.05, ***P < 0.001, vs. ^–/–^. (**I**) Summary of experimental results. Tx, treatment. *P* values reflect pairwise comparisons of means. Values for all data points in graphs are reported in the Supporting Data Values file.

## Version 1 08/28/2025

In-Press Preview

## Version 2 11/17/2025

Electronic publication
